# Symmetry, magnetic transitions and multiferroic properties of *B*-site-ordered *A*_2_Mn*B*′O_6_ perovskites (*B*′ = [Co, Ni])

**DOI:** 10.1107/S2052520624009454

**Published:** 2024-11-08

**Authors:** Jose Luis Garcia-Muñoz, Xiaodong Zhang, Gloria Subías, Javier Blasco

**Affiliations:** a Instituto de Ciencia de Materiales de Barcelona, ICMAB-CSIC Carrer dels Til·lers s/n, Campus de la UAB Bellaterra Catalunya08193 Spain; b Instituto de Nanociencia y Materiales de Aragón (INMA),CSIC-Universidad de Zaragoza, 50009Zaragoza, Spain; c Departamento de Física de la Materia Condensada Universidad de Zaragoza C/ Pedro Cerbuna 12 50009Zaragoza Spain; Brigham Young University, USA

**Keywords:** double perovskites, magnetic structures, multiferroic, frustrated magnetism, magnetic crystallography

## Abstract

The presence of magnetic atoms at the *A* and *B* sites and the coupling between these two spin subsystems in perovskites gives rise to a variety of exciting effects. In particular this coupling attracts interest from the field of novel multiferroic and magnetoelectric oxides. Moreover, magnetic double perovskites presenting cationic order at the *B* sites incorporate an additional modulation that can favor symmetry breaking, multiferroic, magnetoelectric and polar phases. Here, we describe the magnetic structures obtained from neutron diffraction and analyze the symmetry properties of well ordered *A*_2_Mn*B*′O_6_ double perovskites with *A* = Lu, Yb, Tm, Er, Ho, Y, Tb, La_0.5_Tb_0.5_, La and *B*′ = Co or Ni. A rich variety of magnetic orders is formed that have been identified and described, and their symmetry properties are discussed in relation to the multiferroic and magnetoelectric properties of the different compounds.

## Abstract 2

A comparative description is presented of the symmetry and the magnetic structures found in the family of double perovskites *A*_2_Mn*B*′O_6_ (mainly *B*′ = Co and some Ni compounds for comparative purposes).

## 1. Introduction

Rare earth double perovskites of chemical formula *A*_2_*BB*′O_6_ (*A* = trivalent cations as rare earth atoms; *B*,*B*′ = transition metals with different charge and/or different ionic radius) have been investigated in recent years as potential candidates to exhibit multiferroic and magnetoelectric properties. In samples with perfect *B*-site cation order, first principles density functional theory calculations predicted an E-type magnetic ground state for some *BB*′ = NiMn double perovskites such as Y_2_MnNiO_6_ (Kumar *et al.*, 2010 ▸). The so-called E-type is an important type of collinear order characterized by up-up-down-down spin chains along a particular crystallographic direction. Interestingly, it is an example of collinear order that breaks inversion symmetry and can induce magnetoelectric and multiferroic properties. It requires competing magnetic interactions [ferromagnetic (FM) nearest neighbor and antiferromagnetic (AFM) next-nearest neighbor exchanges]. The competition is enhanced in more distorted perovskites, and Monte Carlo calculations predicted that, even in absence of spin-orbit coupling, the coupling of the E-type magnetic modulation to the buckling distortions of oxygen octahedra gives rise to a polar axis perpendicular to the magnetic order direction (Sergienko *et al.*, 2006 ▸). This magnetic ordering along with electric polarization was first observed in orthorhombic HoMnO_3_ (Kimura *et al.*, 2003 ▸). In orthorhombic multiferroic *A*MnO_3_ manganites, the E-type AFM order is in competition with non-collinear spiral orders that also induce ferroelectricity. So, in E-type simple perovskites the exchange striction can modulate the *B*—O—*B* bond angles through O atom displacements. The polarization direction can be different in other E-type scenarios, such as, for example, the quasi-one-dimensional Co–Mn chains of Ca_3_CoMnO_6_, where the new modulation is due to ionic displacements of the magnetic atoms parallel to the direction of the E-type chains (Dong *et al.*, 2015 ▸).

*A*_2_*BB*′O_6_ double perovskites are characterized by the possibility of competitive magnetic interactions. The most important is the superexchange interaction between the atoms located in the *B* position, *J*_*BB*′_, and it usually leads to long-range magnetic orderings at relatively high temperatures. If the *A* position is occupied by magnetic rare earth atoms, exchange interactions usually appear between the atoms of the two sublattices, *J*_*AB*_, with decreasing temperature and at very low temperatures direct interactions arise between the atoms in the *A* position, *J*_*AA*_. Typically, the strength of magnetic interactions follows the sequence, *J*_*BB*′_ > *J_AB_* > *J*_*AA*_. Furthermore, when the structural distortion increases greatly due to the decrease in the size of the rare earth atoms (chemical pressure), the competition between nearest-neighbor and next-nearest-neighbor interactions is enhanced, as mentioned above.

In this report, we will analyze the symmetry-related properties of well ordered *A*_2_Mn*B*′O_6_ double perovskites with *B*′ = Co, Ni, deduced from neutron diffraction experiments. The observed symmetry-related properties have a direct impact on the magnetoelectric and multiferroic behavior of the compounds.

## 2. Experimental

*A*_2_MnCoO_6_ (*A*_2_ = La_2_, LaTb, Tb_2_, Ho_2_, Y_2_, Er_2_, Tm_2_, Yb_2_ and Lu_2_) and *A*_2_MnNiO_6_ (*A*_2_ = Tb_2_) compounds were prepared by solid state reaction from stoichiometric amounts of *A*_2_O_3_, Mn_2_O_3_ and Co_3_O_4_ or NiO as reported elsewhere (Blasco *et al.*, 2014 ▸; Blasco *et al.*, 2017*a* ▸; Blasco *et al.*, 2017*b* ▸; García-Muñoz *et al.*, 2019 ▸; Blasco *et al.*, 2016 ▸). The last sintering step at 1200°C was followed by a slow cooling at 1°C min^−1^ for some *A*_2_MnCoO_6_ (*A*_2_ = La_2_, LaTb) samples. For the rest of the compounds, the last sintering at 1250°C was followed by a very slow cooling at 0.2°C min^−1^. This last cooling step is crucial to improve the cationic ordering at the perovskite *B* site and the oxygen stoichiometry (Dass & Goodenough, 2003 ▸; Kyômen *et al.*, 2004 ▸). The chemical composition of the samples was tested by using the wavelength dispersive X-ray fluorescence spectrometry technique. The cationic composition agreed with the nominal one for all samples within experimental errors.

In order to verify that our specimens were single phase with the expected peaks for a perovskite phase, X-ray powder diffraction (XRD) patterns were collected at room temperature using either a Rigaku D-max or a Siemens D-5000 diffractometer and Cu *K*α radiation. The neutron diffraction experiments were carried out at the high-flux reactor of the Institut Laue Langevin (Grenoble, France) using the D1B (λ = 2.52 Å), D20 (λ = 2.41 Å), and D2B (λ = 1.594 Å) diffractometers. Using helium cryostats, neutron powder diffraction (NPD) patterns were collected at fixed selected temperatures and also following temperature ramps with heating ratios ranging between 0.25 and 2 K min^−1^. Structural and magnetic Rietveld refinements were made using the *FullProf* program (Rodríguez-Carvajal, 1993 ▸). Crystallographic tools from the Bilbao Crystallographic Server (Perez-Mato, 2015 ▸; Perez-Mato *et al.*, 2010 ▸) and the *ISOTROPY* software suite (Campbell *et al.*, 2006 ▸) were also used to perform the symmetry analysis.

Magnetic measurements were carried out between 5 and 300 K using a Superconducting Quantum Interference Device (SQUID) and a Physical Properties Measuring System (PPMS) from Quantum Design. The dielectric characterization of the samples was carried out as a function of temperature and magnetic field in a PPMS employing a home-made coaxial-line inset and an impedance analyzer (Wayne Kerr Electronics 6500B). The electrical characterization was reported earlier in various publications (*e.g.* Blasco *et al.*, 2015 ▸; Blasco *et al.*, 2017*a* ▸; Blasco *et al.*, 2014 ▸; Blasco *et al.*, 2016 ▸).

## 3. Results and discussion

The XRD patterns at room temperature agree with a single-phase perovskite for all samples. All patterns agree with a monoclinic cell with space group *P*2_1_/*n*, typical of double perovskites with atomic ordering at the *B* site coupled with an in-phase rotation around the *c* axis of *B*O_6_ octahedra and an out-of-phase tilt along the [110] direction of the same octahedra (Howard *et al.*, 2003 ▸). The XRD technique has poor sensitivity for detecting the order of nearby atoms in the periodic table. For this purpose, we have used NPD measurements. Refined structural data at room temperature can be consulted in the supplementary information (Table S1). This technique confirms the high degree of Mn/*B*′ ordering (*B*′ = Co, Ni) in all samples prepared with a very slow cooling rate in the last sintering process (Blasco *et al.*, 2017*a* ▸). In this case, the amount of antisite defects (ASDs), *i.e.* the number of Mn and *B*′ atoms exchanging positions is typically ∼5–6% (50% implies random distribution). On the other hand, the two samples obtained with a cooling rate of 1°C min^−1^ present a greater number of ASDs: 18% for La_2_MnCoO_6_ and 42% for LaTbMnCoO_6_, the latter due to the superimposed effect of a strong disorder in the *A* position of the perovskite (Blasco *et al.*, 2014 ▸).

The magnetic order in double perovskites is influenced by several factors such as competitive magnetic interactions, single-ion magnetic anisotropies, external magnetic field, structural disorder or the structural strain produced by the misfit between the size of the cations occupying the two crystallographic sites of the perovskite structure. All these effects determine the different magnetic and magnetoelectric properties in this family of compounds, as it is shown below.

### 3.1. Magnetic orders in the *A*_2_MnCoO_6_ double perovskites

**Figure 1:**
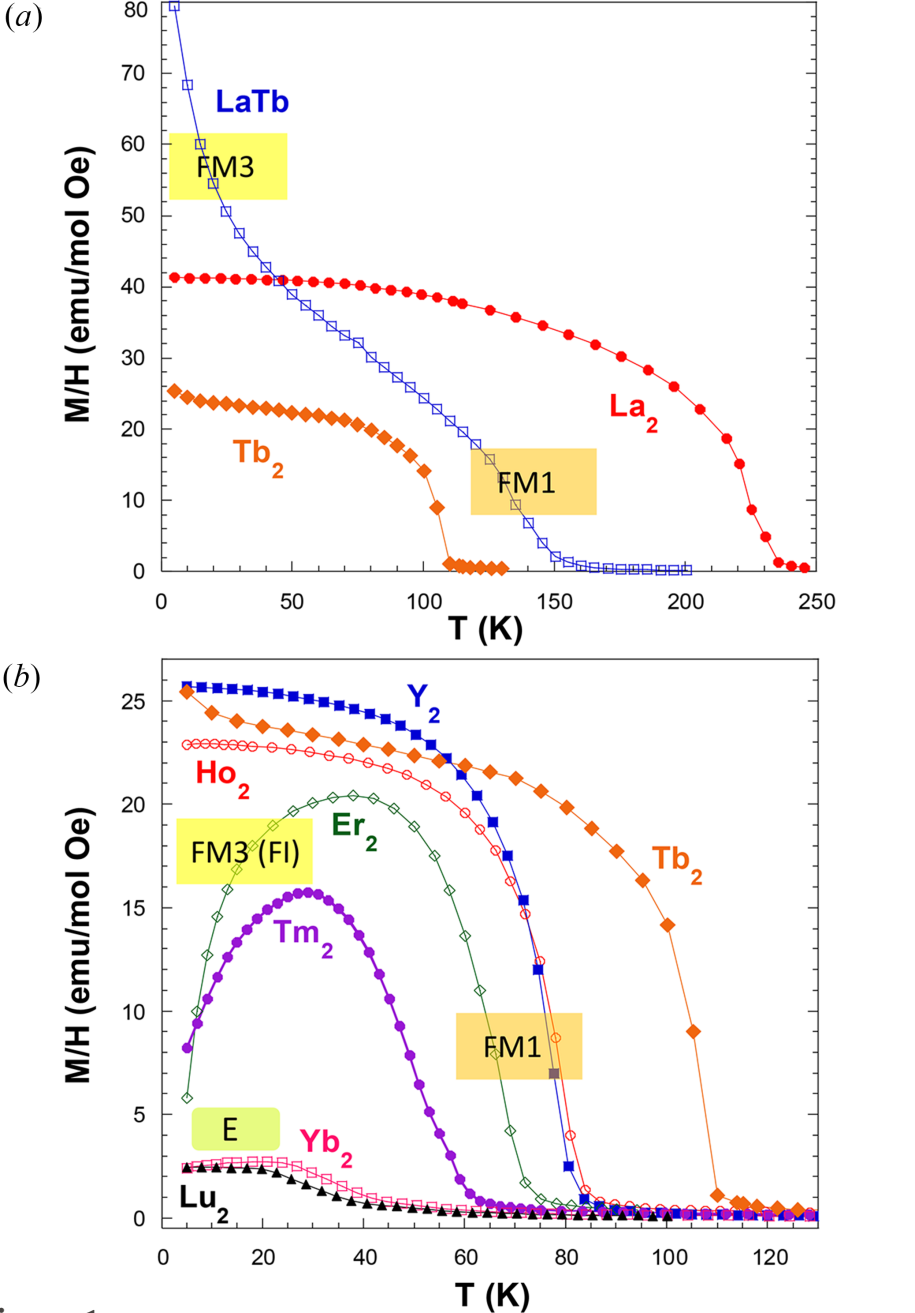
The dc magnetization under a field of 100 Oe in field cooled conditions for (*a*) La- and Tb-based compounds and (*b*) *A*_2_MnCoO_6_ samples with the *A* atom indicated for each curve. For comparison purposes, Tb_2_MnCoO_6_ is included in both panels. Labels indicate different types of magnetic ordering (see explanation in the text).

Fig. 1 ▸ shows the temperature dependence of dc magnetization, *M*(*T*), for *A*_2_MnCoO_6_ compounds. The measurements were taken in a magnetic field of 100 Oe after field cooling. Clear magnetic transitions are observed for all samples but their transition temperatures decreases as the *A*-atom size decreases. A smaller size of the *A* atoms enhances the Mn(Co)O_6_ tilts and weakens the ferromagnetic Mn—O—Co superexchange interaction. Ferromagnetic-like transitions are observed in the *M*(*T*) curves for *A*_2_MnCoO_6_ compounds with *A*^3+^ radius (Shannon, 1976 ▸) between La^3+^ [*r*_La_^3+^(IX) = 1.216 Å] and Tm^3+^ [*r*_Tm_^3+^(IX) = 1.052 Å]. For smaller *A* atoms (Yb, Lu), the *M*(*T*) curves are more typical of an antiferromagnetic ordering. In our previous studies we have disclosed several magnetic structures in the *A*MnCoO_6_ series (Blasco *et al.*, 2017*a* ▸; Blasco *et al.*, 2016 ▸). The paramagnetic phase can be described in terms of the *P*2_1_/*n*1′ gray group whose properties are indicated below.

*The gray group *P*2_1_/*n*1′.* In this phase [No. 14.76 in BNS notation (Belov *et al.*, 1957 ▸)], and assuming a perfect 3*d* cationic ordering, *B* and *B*′ cations occupy, respectively, the Wyckoff positions 2*c* (0, ½, 0) and 2*d* (½, 0, 0). *A* cations are placed at 4*e* (*x*, *y*, *z*), as the three independent O sites. For simplicity, the *P*2_1_/*n* setting chosen for the parent cell in the compounds described in Tables S1 and S2 is that whose transformation to the standard setting (*P*2_1_/*c*1′) is (**a**, **b**, −**a**+**c**; 0, 0, 0) [or also (**c**, **b**, −**a**−**c**; 0, 0, 0)]. Unlike the *B*,*B*′-site symmetries (−1), the symmetry of the *A* positions does not contain the inversion center and is simply 1. The decomposition of the magnetic representation into irreducible representations (irreps) of the gray group *P*2_1_/*n*1′ (**k** = 0) is shown in Table 1 ▸, the dimensions of the irreps of the little group being all 1.

#### 3.1.1. Ferromagnetic order of *B* sublattices (**FM1** phase)

**Figure 2:**
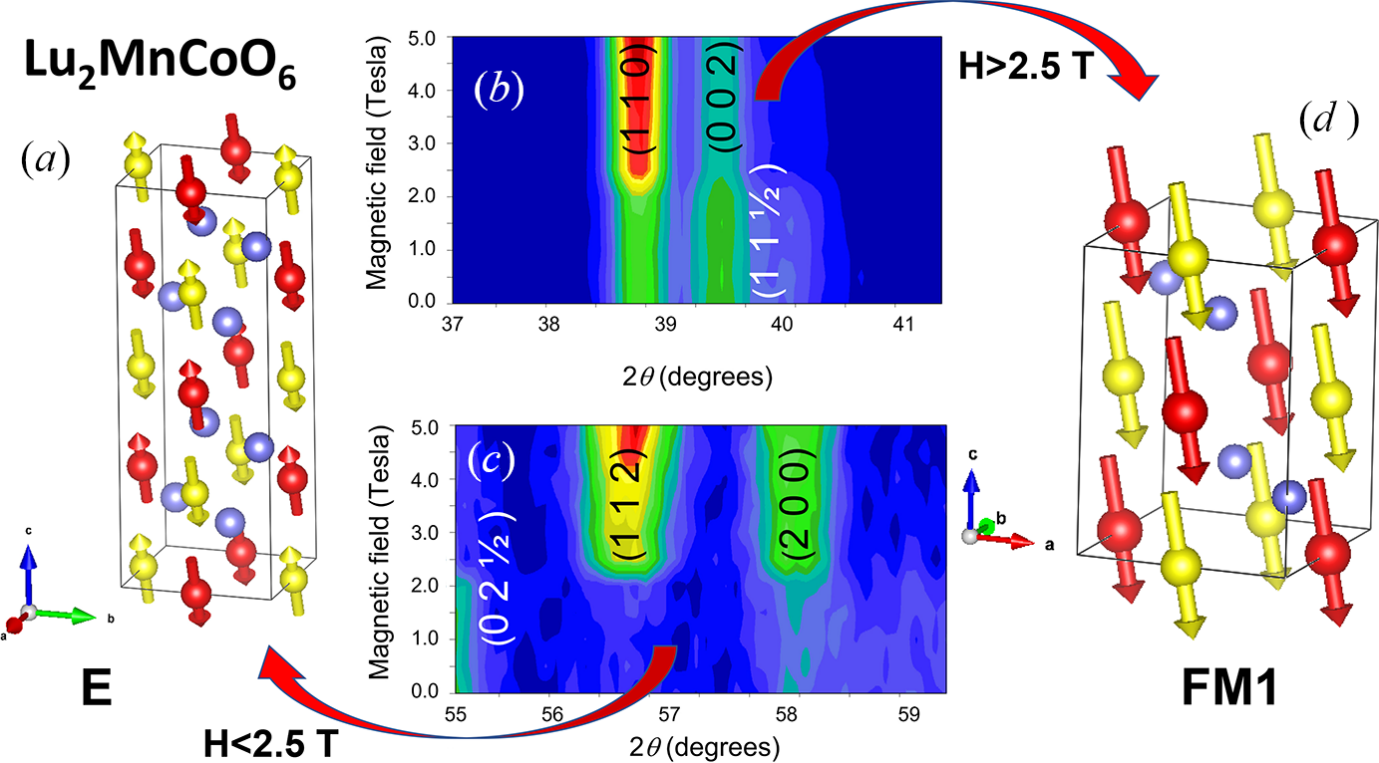
(*a*) Magnetic structure of Lu_2_MnCoO_6_ at 4 K for low external fields (*H* < 2.5 T). (*b*) and (*c*) Details of the field dependence of neutron intensities collected at D1B for Lu_2_MnCoO_6_ showing both the vanishing of superstructure E-type peaks and the enhancement of FM peaks with increasing magnetic field. (*d*) Magnetic structure of Lu_2_MnCoO_6_ at 4 K for high external fields (*H* > 2.5 T). Red and yellow balls (arrows) stand for Mn and Co atoms (moments), respectively. Oxygen atoms have been removed for the sake of clarity.

A long-range FM ordering is observed for *A*_2_MnCoO_6_ compounds (*A* size between La and Tm) and, according to the Goodenough–Kanamori superexchange rules (Goodenough, 1958 ▸; Kanamori, 1959 ▸), it is ascribed to the FM Mn^4+^–O–Co^2+^ superexchange interaction as Mn^4+^ ion has empty 3*d*–*e*_*g*_ orbitals while they are half-filled for high-spin Co^2+^ cations in an octahedral coordination. The crystal structure adopts the space group *P*2_1_/*n* (No. 14, standard group *P*2_1_/*c*). The non-standard description is often preferably used by scientific community because the β angle is closer to 90° and allows an easier association with the undistorted cubic structure. The magnetic peaks can be well reproduced by the maximal magnetic subgroup *P*2_1_′/*n*′ (No. 14.79 in BNS notation) that keeps unchanged the dimensions of the magnetic cell (the magnetic propagation vector is ***k*** = 0, see Table 2 ▸). The setting used in Table 2 ▸ to describe the magnetic symmetry corresponds to the parent setting, and transforms to the standard basis *P*2_1_′/*c*′ through the transformation (**a**, **b**, −**a**+**c**; 0, 0, 0). This magnetic arrangement [*P*2_1_′/*n*′ (**a**, **b**, −**a**+**c**; 0, 0, 0)] transforms as the magnetic irrep mGM2+ respect to the parent nuclear structure with *P*2_1_/*n* symmetry (see Table 3 ▸). This one-dimensional magnetic irrep (mirrep) has three potential modes, two of them allow a ferromagnetic alignment of the Mn^4+^ and Co^2+^ moments along the *ac* plane. The third mode allows an AFM arrangement of the moments along the *b* axis (*A* type). In almost all *A*_2_MnCoO_6_ compounds, this third mode is inactive and the magnetic order is ferromagnetic with the largest component along the *c* axis. This is a very common magnetic phase in this family of compounds and hereafter, we denote it as **FM1** phase. A representative description of this type of order can be seen in Fig. 2 ▸(*d*) and its description for Ho_2_MnCoO_6_ is summarized in Tables 2 ▸ and 3 ▸. Similar tables for the rest of the compounds can be found in the supporting information. The only exception known at the moment (Blasco *et al.*, 2016 ▸) corresponds to Y_2_MnCoO_6_, which shows a small AFM canting along the *b* axis as can be seen in the supporting information.

#### 3.1.2. Antiferromagnetic order of *B* sublattices (E phase)

**Figure 3:**
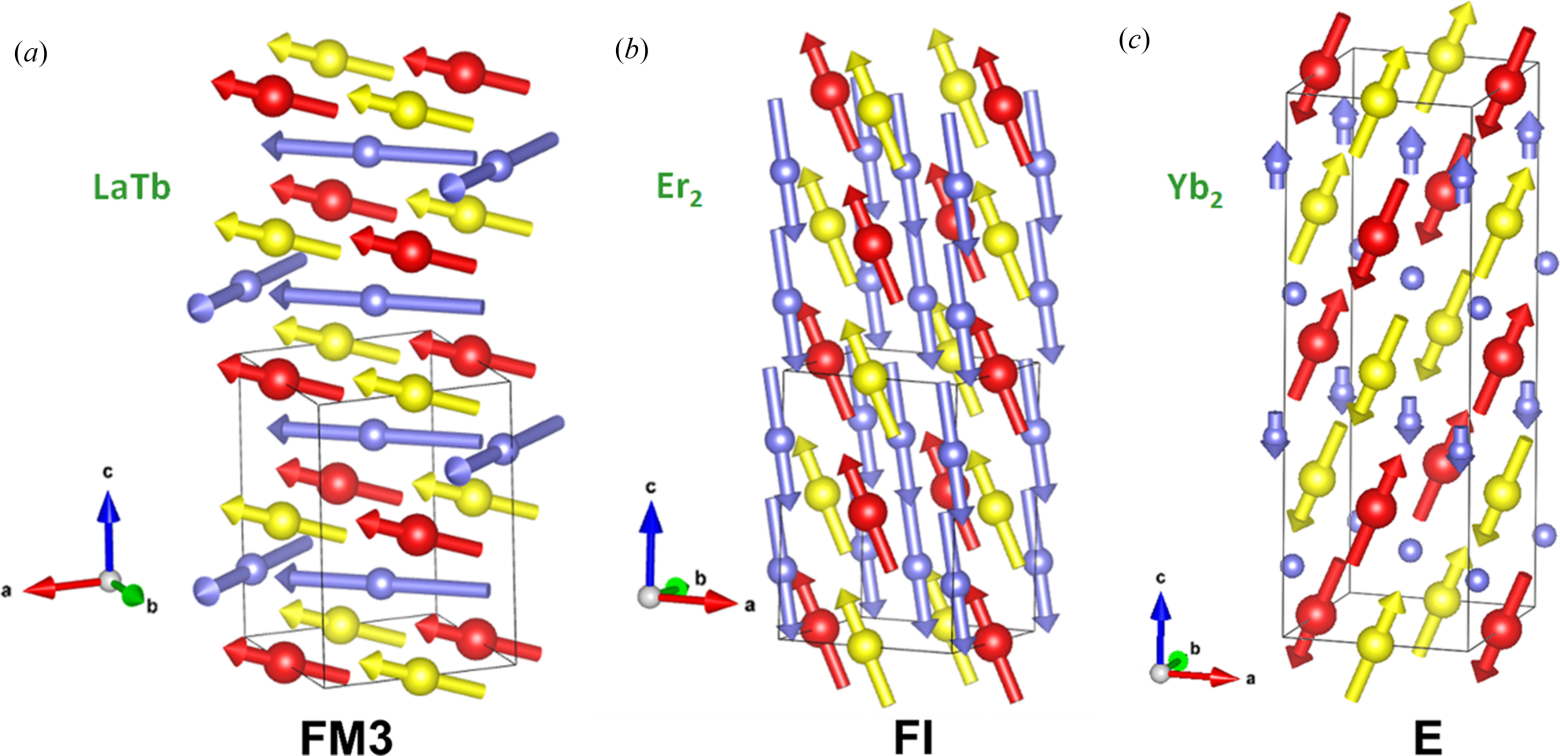
Magnetic structures at 2 K of (*a*) LaTbMnCoO_6_, (*b*) Er_2_MnCoO_6_, and (*c*) Yb_2_MnCoO_6_. Red, yellow and purple balls (arrows) stand for Mn, Co and rare earth atoms (moments), respectively. Oxygen atoms have been removed for the sake of clarity.

For heavier rare earth atoms (*A* = Yb or Lu), the increase in structural distortion produces a large decrease in the Mn—O—Co bond angle causing the FM interaction to nearest neighbors to compete with the AFM interaction with the next nearest neighbors. This causes the magnetic ground state to change and it is no longer FM but AFM of E-type, *i.e.* displaying up-up-down-down (↑↑↓↓) spin chains along a specific crystallographic direction [see Fig. 2 ▸(*a*)]. The magnetic peaks can be indexed using the propagation vector **k** = (0, 0, ½) (*P*2_1_/*n* setting), and the refinements indicate that Mn and Co moments are mainly aligned along the *c* axis (Blasco *et al.*, 2015 ▸). The magnetic space group (MSG) in both *A*_2_MnCoO_6_ compounds (Yb and Lu) is *P*_*a*_2_1_ (No. 4.10) and, using the parent-like setting (**a**, **b**, 2**c**; 0, 0, 0) a transformation to the standard setting *P*_*a*_2_1_ is given by (−**c**, **b**, **a**; ¼, 0, 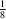) [or (2**a**−**c**, **b**, **a**; −¼, 0, 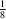)] (Table 4 ▸). The primary mirrep mA1 is associated with the *k*-point (−1, 0, ½), equivalent to (0, 0, ½), of the non-standard parent *P*2_1_/*n* (**a**, **b**, −**a**+**c**; 0, 0, 0) structure. There are three active modes for the perovskite *B* sites in mA1. Two of them permit the spins of the up-up-down-down chains to be parallel to the *ac* plane while the third mode allows a *b* axis spin component. Tables 4 ▸ and 5 ▸ describe the E-type magnetic structure of Lu_2_CoMnO_6_, represented in Fig. 2 ▸(*a*). As can be seen, the spins in the chains are arranged in the *ac* plane with the largest component along the *c* axis. Although it is allowed by the symmetry the contribution of the third mode was below the detection limit of the powder diffraction experiment. For that reason its amplitude has been set to zero (Table 5 ▸). Similar ordering is observed in the Yb_2_MnCoO_6_ compound [Fig. 3 ▸(*c*)] although in this case there is also a partial order of the Yb sublattice as we will see in the next section. It is noteworthy that this magnetic space group is polar and allows a ferroelectric ordering along the monoclinic unique axis. The polar axis of the point symmetry 2.1′ (No. 3.2.7) is along **b**, and hence compatible with spontaneous *P*_*y*_ electric polarization. Another property of this magnetic order is its instability in the presence of an external magnetic field, giving rise to metamagnetic transitions (Blasco *et al.*, 2015 ▸). The application of a magnetic field produces the spin flop of half of the chain moments, to yield a linear FM ordering similar to that of the *A*_2_MnCoO_6_ compounds at zero field (**FM1**). Fig. 2 ▸ shows the field dependence of representative parts of the NPD patterns at 4 K for Lu_2_CoMnO_6_. At zero field, it shows the presence of E-type superstructure peaks that vanish with increasing the external magnetic field and accordingly, the net FM contribution is enhanced, see Figs. 2 ▸(*b*) and  ▸2(*c*). The E-type spin ordering breaks the inversion center of the crystal cell and a finite ferroelectric polarization perpendicular to the E-chains direction is allowed. Then, at the same temperature that E-type magnetic order develops, an anomaly is observed in the dielectric permittivity that indicates the occurrence of the ferroelectric phase (Blasco *et al.*, 2017*a* ▸). This dielectric anomaly vanishes in the presence of an external magnetic field, as can be seen in the supporting information (Fig. S1). The external magnetic field produces a simultaneous spin reorientation giving rise to the conventional FM phase observed in the previous *A*_2_MnCoO_6_ compounds. This metamagnetic transition leads to the negative magnetocapacitance shown in Fig. S1. Fig. 2 ▸ also shows the two types of magnetic ordering for Lu_2_MnCoO_6_, the E-type order in the absence of a magnetic field and the FM order (denoted as **FM1**) in the presence of an external field.

#### 3.1.3. Polarization and magnetic ordering of the *A* site

A close inspection of Fig. 1 ▸ reveals significant differences in the low-temperature behavior of the *M*(*T*) curves for ferromagnetic *A*_2_MnCoO_6_ compounds. Firstly, the compounds with nonmagnetic *A* atoms (*A*_2_ = La_2_, Y_2_) present an almost constant magnetization evolution at low temperature. Instead, some samples with magnetic *A* atoms present a strong rise in magnetization at low temperature (*A*_2_ = LaTb) while others suffer a strong decrease (*A*_2_ = Er_2_, Tm_2_). This behavior must be ascribed to the mutual polarization between the two sublattices of magnetic atoms and the competition among the *J*_*AB*_, *J*_*BB*′_ and *J*_*AA*′_ interactions. For Er- and Tm-based samples, the changes in the neutron magnetic scattering can be accounted for only by including the magnetic contribution from the Tm(Er) sublattice. The refinements indicate that either Tm^3+^ (Blasco *et al.*, 2017*a* ▸) or Er^3+^ (Blasco *et al.*, 2017*b* ▸) moments are antiferromagnetically coupled to the Mn–Co sublattice, revealing a negative *J*_*AB*_ interaction for these compounds with heavy rare earth atoms. The arrangement of the rare earth follows the same mirrep (mGM2+) as can be seen in the Tables S5 (Er) and S7 (Tm) so the magnetic group remains *P*2_1_′/*n*′. The rare earth sublattice is not completely polarized but the main component of this antiferromagnetic arrangement is found along the *c* axis as seen in Fig. 3 ▸ and we denote this ferrimagnetic structure as **FI**. The ordering is not fully collinear disclosing a competition between the magnetic interaction and the rare earth anisotropy. Another distinct example of interaction between the two magnetic sublattices occurs in the LaTbMnCoO_6_ compound (Blasco *et al.*, 2014 ▸). In this case, the interaction *J*_*AB*_ is positive leading to a ferromagnetic coupling between the two sublattices. However, the strong anisotropy of Tb^3+^ cations and the constrain of local point symmetry give rise to a confinement of Tb^3+^ moments in the *ab* plane. Its arrangement is similar to that found in TbFeO_3_ at 4.2 K (Bertaut *et al.*, 1967 ▸) and is denoted as *F*_*x*_*C*_*y*_ in Bertaut’s notation (Bertaut, 1968 ▸). This means that the FM component of the Tb sublattice is directed along the *a* axis and the *J*_*AB*_ interaction is so strong that it forces the moments of the Mn(Co) sublattice to tilt in that direction as seen in the Fig. 3 ▸. We denote this type of ordering involving *A* and *B* moments as **FM3** because of its analogy with the same type of order observed in Tb_2_MnNiO_6_ (see below). The ordering of the two sublattices is compatible with the same mirrep (mGM2+) as can be seen in Table S6. Interestingly, as Tb concentration increases, a third interaction, *J*_*AA*_, begins to gain importance in Tb_2_MnCoO_6_. The direct Tb–Tb interaction, stronger at low temperatures, usually induces an *A*_*x*_*G*_*y*_-type order in related systems (Mareschal *et al.*, 1968 ▸; Cuartero *et al.*, 2013 ▸). The strong competition between *J*_*AB*_ and *J*_*AA*_ leads to magnetic frustration in the Tb sublattice and neutron patterns at 2 K only shows the FM ordering of Mn–Co sublattice (see Table S3), mainly along *c* axis, and a diffuse scattering typical of short-range ordering of the Tb sublattice (Blasco *et al.*, 2014 ▸). A similar effect occurs in the Ho_2_MnCoO_6_ sample, where diffuse scattering associated with the short-range ordering of Ho^3+^ moments is also observed at low temperature (Blasco *et al.*, 2017*a* ▸).

Finally, the polarization of the rare earth sublattice also shows some interesting features in the E-type magnetic structure of Yb_2_MnCoO_6_ (see Table S8). Unlike Lu^3+^, Yb^3+^ is magnetic and experiences the internal field produced by the E magnetic arrangement of the Mn–Co sublattice (*T*_N_ = 50 K). At sufficiently low temperatures (*T* < 15 K), the Yb atoms split in two different orbits in the magnetic cell with *P*_*a*_2_1_(−**c**, **b**, **a**; ¼, 0, 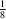) [also (2**a**−**c**, **b**, **a**; −¼, 0, 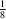)] symmetry. In the Yb1 orbit, the Yb atoms experience a strong internal field from the FM arrangement of neighboring Mn/Co moments, which prevents the AF coupling between Yb atoms and keeps them disordered. In the other Yb orbit (Yb2) instead, the AFM arrangement of neighboring Mn/Co moments cancels the internal field at the Yb position and the magnetic Yb–Yb interaction produces a long-range AFM ordering in these atoms below 15 K as can be seen in the Fig. 3 ▸(*c*) (Blasco *et al.*, 2017*a* ▸). The magnetic space group is preserved below the Yb ordering temperature. Fig. S1 illustrates the anomalous dielectric permittivity concurrent with the formation of the E-type phase (*P_a_*2_1_) and its response to external magnetic fields. This phase also shares polar magnetic point symmetry (21′) with Lu_2_MnCoO_6_.

### 3.2. Tb_2_MnNiO_6_: polar ferrimagnetic ground state by incompatibility of *A* and *B* sites magnetic symmetries

**Figure 4:**
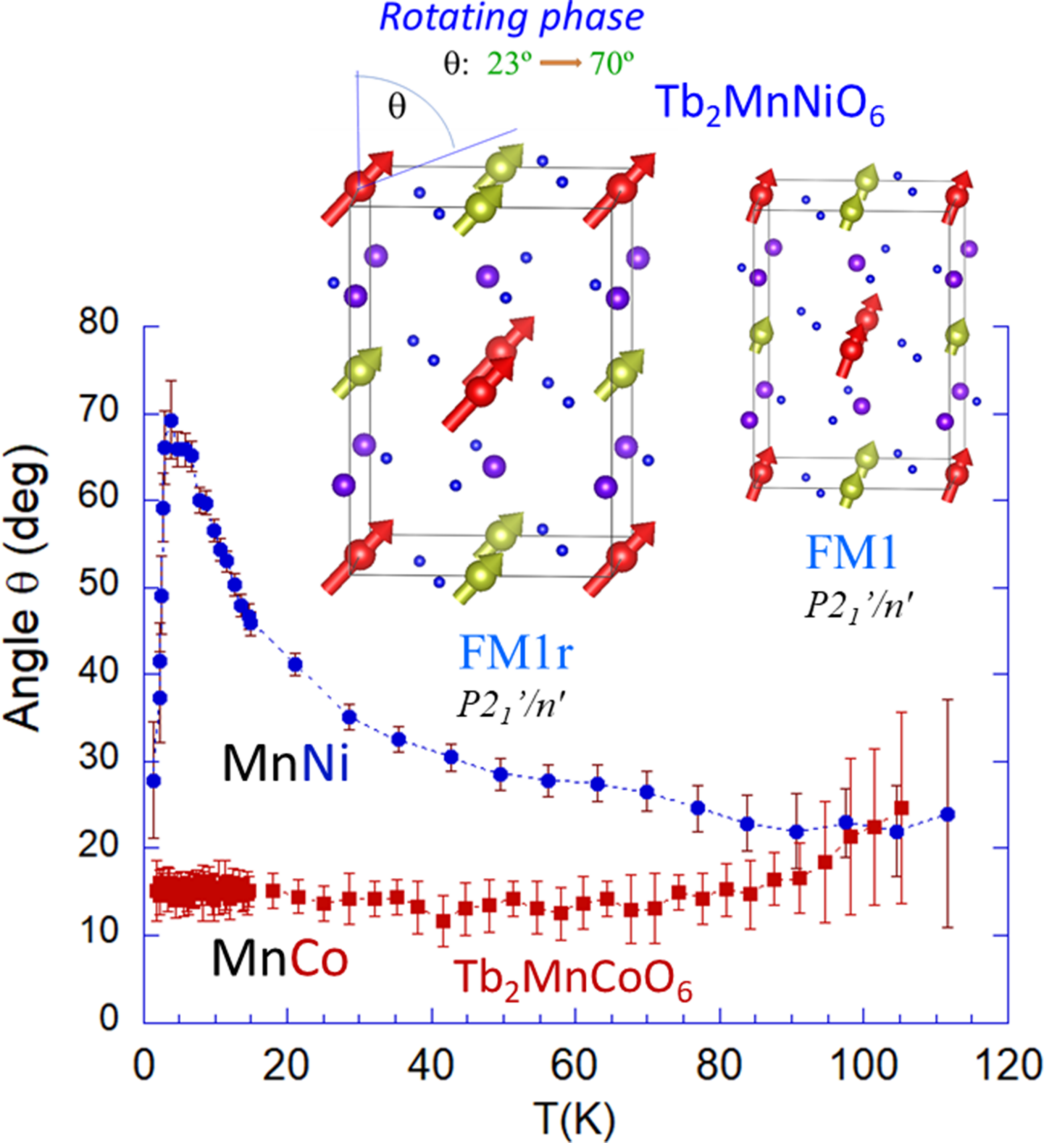
Tb_2_Mn*B*′O_6_ (*B*′ = Ni and Co). Comparison of the direction of 3*d* moments (θ) and temperature evolution. Inset: **FM1** and **FM2** (= **FM1r** [rotating]) magnetic phases of Tb_2_MnNiO_6_ (*P*2_1_′/*n*′).

In this section we present a comparison of the magnetic transitions and symmetries in the isostructural perovskites Tb_2_Mn*M*O_6_ with *M* = Ni and Co, having monoclinic *P*2_1_/*n* distortion and almost perfect ordering of the Mn^4+^/*M*^2+^ 3*d* metals. As for Tb_2_MnCoO_6_, the structure of Tb_2_MnNiO_6_ is detailed in Table S1 using the nonstandard *P*2_1_/*n* setting. Our non-standard description of the parent structures is given in the non-conventional setting (−**a**−**c**, **b**, **a**; 0, 0, 0) of the *P*2_1_/*c* group (No. 14). As in Tb_2_MnCoO_6_, the magnetic transitions in Tb_2_MnNiO_6_ are all driven by zone-center modes (***k*** = 0) of the parent phase. Below the paramagnetic *P*2_1_/*n* phase (MSG * P*2_1_/*n*1′), up to four ordered regimes can be distinguished in its magnetic evolution under cooling.

(*a*) **FM1** phase (120 K, 70 K). MSG *P*2_1_′/*n*′ (**a**, **b**, −**a**+**c**; 0, 0, 0). The ferromagnetic transition temperature (*T*_C1_ ∼ 120 K) is higher than in the homologous Tb_2_MnCoO_6_ compound (∼100 K) (Blasco *et al.*, 2016 ▸), and magnetic intensities agree with the maximal subgroup *P*2_1_′/*n*′ (No. 14.79 in BNS notation) (García-Muñoz *et al.*, 2019 ▸). As in previous *A*_2_MnCoO_6_ perovskites this ferromagnetic ordering transforms as the irrep mGM2+ [compatible with a magnetic arrangement of the *F*_*x*_*C*_y_*F*_*z*_ type in the Bertaut’s notation (Bertaut, 1968 ▸)]. The m_*y*_ component was not detected in our measurements and the observed FM spins are confined in the *ac* plane but almost aligned parallel to the *c* axis of the perovskite [Mn and Ni moments make an angle θ ≃ 23 (3)° with *c*].

(*b*) **FM2** phase [70 K, 15 K]. MSG *P*2_1_′/*n*′ (**a**, **b**, −**a**+**c**; 0, 0, 0). If we continue to cool, the neutron intensities disclose a new regime where the FM *P*2_1_′/*n*′ order persists but, in parallel, the direction of the FM Mn^4+^/Ni^2+^ moments continually rotates towards the *a* axis upon cooling (see Fig. 4 ▸ and Table 6 ▸), so that we have named this ‘*rotating* FM phase’ **FM2** = **FM1r** (inset of Fig. 4 ▸). In this regime the single active mGM2+ irrep is preserved. Mn and Ni keeps the collinear order and the magnetization forms an angle θ with the *c* axis that increases from 23 (3)° (75 K) up to 46 (2)° (15 K). The Tb sublattice plays a key role on the *rotating* phase and on the evolution followed by the tilting angle θ. The rotating magnetization is surely driven by a ferromagnetic Tb–(Mn, Ni) interaction (*J*_Tb–*M*_ > 0) that attracts the 3*d* moments towards the *ab* plane. Short-range ferromagnetic correlations between Tb moments were perceived by neutron diffraction below around ≃70 K. In fact, Fig. 4 ▸ reveals that the magnetization rotates ∼50° in the short interval 5–70 K, although partial long-range ordering of Tb moments was only detected below *T*_N3_ ≃ 15 K.

(*c*) **FM3** phase (15 K, 3.7 K). MSG *P*2_1_′/*n*′ (**a**, **b**, −**a**+**c**; 0, 0, 0). The ordering of terbium moments in Tb_2_MnNiO_6_ involves two successive magnetic configurations. First there is an intermediate arrangement in which the overall *P*2_1_′/*n*′ symmetry is preserved and terbium moments become partially polarized with the help of the FM exchange field created by Mn/Ni sites. The refined magnetic configuration at 6 K is given in Table 7 ▸. Tb moments preserve a single independent orbit and adopt the symmetry dictated by the mGM2+ irrep, yielding two identical Tb magnetic layers parallel to *ab* (at *z* ≃ 0.25 and *z* ≃ 0.75) that are connected by inversion symmetry ({−1 | 0, 0, 0}). A configuration analogous to the ground state in LaTbMnCoO_6_ [Fig. 3 ▸(*a*) and Table S6]. Tb moments are confined in the *ab* plane and adopt the permitted Bertaut *F*_*x*_*C*_*y*_*F*_*z*_ configuration with *F*_*z*_ = 0 within our detection limit. The common *F*_*x*_ component points to a ferromagnetic interaction between 4*f* and 3*d* ions. Since this **FM3** magnetic structure conserves the spatial inversion its point magnetic symmetry [2′/*m*′ (No. 5.5.16)], is *nonpolar*.

(*d*) **FM4** phase (*T* < 3.7 K). MSG *P*2_1_′ (**a**, **b**, **c**; ¼, 0, ¼). Upon further cooling, when temperature decreases below *T*_N4_ ≃ 3.7 K, a new magnetic transition reveals the magnetic ground state of Tb_2_MnNiO_6_. In this new phase (**FM4**, described in Table 8 ▸) the inversion symmetry operation connecting Tb magnetic ions at the two different TbO_2_ layers is lost. As a consequence, during the *P*2_1_′/*n*′ to *P*2_1_′ transformation the only Tb position in *P*2_1_′/*n*′ is split to give two independent Tb sublattices below the transition. Each of the Tb1 and Tb2 orbits corresponds to a different layer in the unit cell. Only the positions of the 3*d* metals are not split (its site symmetry changes from −1 to 1). In this ground phase each one of the three oxygen sites (O1, O2 and O3) splits in two orbits to give six independent oxygen positions (Table 8 ▸).

**Figure 5:**
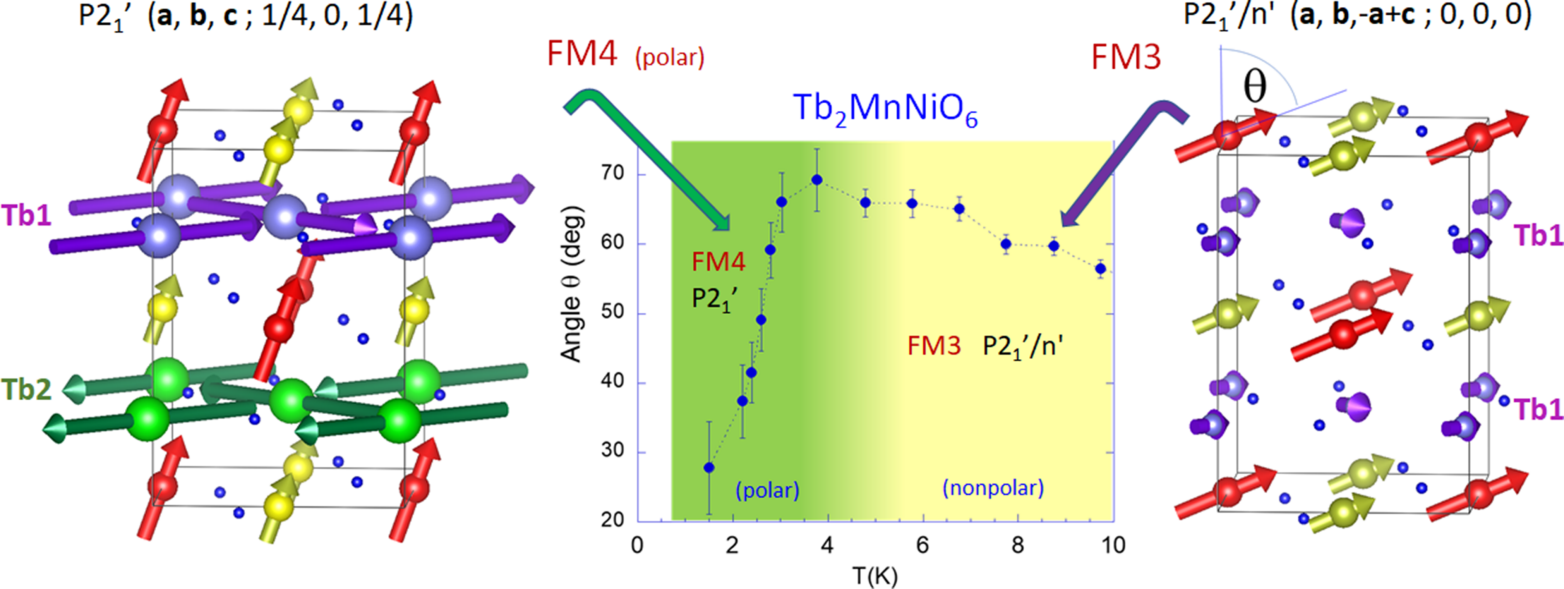
Tb_2_MnNiO_6_: polar/non-polar magnetic transition (**FM4**/**FM3**). Inclination angle (θ) of 3*d* moments (center). Magnetic structures: (left, **FM4**) *P*2_1_′ (**a**, **b**, **c**; ¼, 0, ¼) and (right, **FM3**) *P*2_1_′/*n*′ (**a**, **b**, −**a**+**c** ; 0, 0, 0).

As shown in Fig. 5 ▸, the Tb–Tb interactions transform the *F*_*x*_*C*_*y*_ magnetic configuration of Tb moments (**FM3**) into *A*_*x*_G_*y*_ (**FM4**). Tb^3+^ moments in the same layer form angles close but below 90°. As occurs in other terbium perovskites, the planar magnetic arrangement of Tb in the **FM3** and **FM4** phases of Tb_2_MnNiO_6_ reveals the Ising nature of the terbium moments derived from the lowest crystal-field energy levels. The electronic configuration of Tb^3+^ ions (4*f*^8^) and its non-Kramers nature entails a very large local anisotropy in low-symmetry sites. Typically, an accidental quasi-doublet gives it Ising-like properties (Novák *et al.*, 2013 ▸; Gruber *et al.*, 2008 ▸). The drop in the exchange field produced by the flipping of Tb moments in one of the two layers is followed by a sudden strong reorientation of the FM 3*d* moments towards the *c* axis (Δθ ≃ −40°). The latter is illustrated in Fig. 5 ▸ that displays the evolution of the magnetization direction (θ) below 10 K and a view of the **FM4** and **FM3** magnetic structures of Tb_2_MnNiO_6_. In Fig. 4 ▸ we have compared the distinct evolution of the magnetization directions θ(*T*) in Tb_2_MnNiO_6_ and Tb_2_MnCoO_6_ in all the range below the paramagnetic boundary.

**Figure 6:**
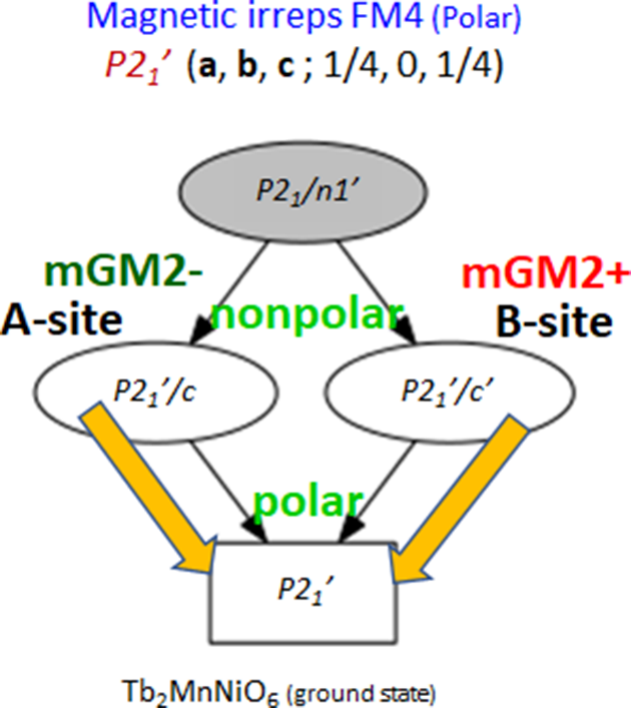
Tb_2_MnNiO_6_: active primary magnetic irreps in the polar magnetic ground state [*P*2_1_′ (**a**, **b**, **c**; ¼, 0, ¼)]. The overlay of the non-polar mGM2+ (*B* sites) and mGM2− (*A* sites) modes generates the polar *P*2_1_′ MSG and causes a severe reorientation (Δθ ≃ 50°) of the magnetization.

Regarding the magnetic distortion modes, in Table 9 ▸ we describe the main mirreps and their associated magnetic modes active at the ground state of Tb_2_MnNiO_6_ (**FM4**), with respect to its paramagnetic phase. In addition, Fig. 6 ▸ illustrates that (according to Table 9 ▸) two primary magnetic irreps of the parent group (mGM2+ and mGM2−) are the responsible for the magnetic symmetry of Tb_2_MnNiO_6_ below *T*_N4_ ≃ 4 K. First, the ferromagnetic arrangement of the two 3*d* (Mn/Ni) sublattices, is giving by three one-dimensional magnetic mGM2+ modes, one of which has null amplitude or below our detection limit (*F*_*y*_ = 0, Table 9 ▸). The isotropy subgroup of these ferromagnetic modes is *P*2_1_′/*n*′. In the **FM3** intermediate magnetic regime of Tb_2_MnNiO_6_, 4*f* moments become partially polarized by the exchange field from 3*d* atoms and Mn, Ni and Tb all transform as the irrep mGM2+ (**k** = 0) giving to identical TbO_2_ magnetic layers (Table 3 ▸). The three mGM2+ modes for Tb have non-null amplitude and isotropy subgroup *P*2_1_′/*n*′. Finally, in the **FM3**-to-**FM4** transformation the Tb^3+^ single-ion anisotropy and Tb^3+^–Tb^3+^ energy terms force the substitution of the mGM2+ distortion modes in the Tb sublattice by the mGM2− magnetic modes that split in two orbits the Tb magnetic sites. If the mGM2+ irrep at the zone-center generates the isotropy subgroup *P*2_1_′/*n*′ (No. 14.79) (now from *B* sites only), the new mGM2− magnetic distortion occurring at the *A* sites of the perovskite induces *P*2_1_′/*n* (No. 14.77) symmetry. So, the final symmetry of the **FM4** phase is the MSG *P*2_1_′ (4.9), the overlay (common maximal subgroup) of these two different isotropy subgroups associated to, respectively, the magnetic arrangement in the A and B sublattices. Notice that although, separately, mGM2+ and mGM2− modes are non-polar, the final symmetry of the ground state is *polar* (magnetic point symmetry 2′, No. 3.3.8) and permits the appearance of ferroelectricity with spontaneous polarization parallel to the unique axis *b*. Moreover, as shown in Fig. 5, there is a strong reorientation of the magnetization direction (θ) concurrent with the *P*2_1_′/*n* ↔ *P*2_1_′ phase transition. If the ground state (**FM4**) is ferroelectric due to magnetic trilinear coupling, a cross-correlation between the voltage (magnetic field direction) and the direction of magnetization (electric polarization) is anticipated.

The above properties related with phases **FM2**, **FM3** and **FM4** of Tb_2_MnNiO_6_ were not observed in the analogous Tb_2_MnCoO_6_ compound, which displays a single ferromagnetic **FM1** ordered phase with *P*2_1_′/*n*′ symmetry down to 1.5 K. The large single-ion anisotropy term of divalent cobalt anchors Co^2+^ FM spins almost perpendicular to the *xy* Tb layers, regardless of temperature below *T*_N_ = 100 K. This does not favor the appearance of the rotational **FM2** (= **FM1r**) phase nor the **FM3**. Moreover, the **FM4** order and the non-polar/polar transition were not observed in Tb_2_MnCoO_6_ down to our base temperature.

## 4. Conclusions

We have reported an exhaustive description of the magnetic structures found in the rare-earth double perovskites *A*_2_Mn*B*′O_6_ (*A* = rare earth or Y: *B*′ = Co or Ni). The main magnetic interaction present in these compounds (Mn–O–*B*′ superexchange) gives rise to a FM arrangement in the *ac* plane that adopts the *P*2_1_′/*n*′ magnetic space group. As the *A*-atom size decreases in *A*_2_MnCoO_6_ series, this FM interaction weakens and competes with the next-nearest-neighbor AFM interaction causing an E-type magnetic ordering for Yb- and Lu-based samples [**k** = (0, 0, ½)]. The magnetic structure adopts the *P*_*a*_2_1_ MSG with up-up-down-down spin chains along the *c* axis. This magnetic ordering (*T*_N_) breaks the inversion center and electrical polarization along the monoclinic unique axis, perpendicular to the magnetic direction, is permitted. Therefore, an anomaly in the dielectric permittivity is observed at *T*_N_ but the presence of a sufficiently strong magnetic field induces a spin flop and these distorted compounds adopt the same magnetic structure as the rest of the series at zero field. In the case of magnetic *A* atoms, the magnetic interaction between *A* and *B* sublattices is observed at temperatures lower than the FM ordering of the *B* sublattices. This interaction between sublattices is positive for samples with diluted Tb and due to its strong anisotropy, a change in orientation of the Mn–Co sublattice moments occurs, approaching the *a* axis. In other cases (Er or Tm), the coupling between both sublattices is negative and non-collinear ferrimagnetic orderings are observed in the *ac* plane with the main component along the *c* axis. All these rare earth arrangements can be described in the same magnetic space group, *P*2_1_′/*n*′. Finally, for Tb- and Ho-based Mn/Co samples, the strong *J*_*AA*_ interaction competes with the other two interactions and does not favor the coupling between *A* and *B* sublattices. Mn and Co keep the FM alignment, mainly along the *c* axis while only diffuse magnetic scattering is observed down to 2 K from frustrated moments in the *A* sublattice. The large single-ion anisotropy term of divalent cobalt anchors the 3*d* FM spins almost perpendicular to the Tb layers in the *ab* plane. This configuration generates an internal exchange field that precludes the successive magnetic phases observed in Tb_2_MnNiO_6_, where Ni^2+^ presents a much smaller single-ion anisotropy respect to Co^2+^ ions. In the ground state of Tb_2_MnNiO_6_ the *A* and *B* sublattices adopt, respectively, two different non-polar magnetic irreps whose intersection generates a polar *P*2_1_′ magnetic symmetry compatible with spontaneous *P_y_* polarization assisted by magnetic trilinear coupling. Increasing temperature, a ferrimagnetic Tb-site ordering can be stabilized in Tb_2_MnNiO_6_, canceling the splitting of the Tb sublattices by recovering the inversion symmetry, with the help of a severe spin reorientation (Δθ ≃ 50°) of the ferromagnetic Mn/Ni moments, which align almost parallel to the *ab* plane. In this material the direction of the magnetization could be used as a lever to switch the polar/non-polar (ferroelectric/antiferroelectric) transformation.

## Supplementary Material

CIF and mCIF files. DOI: 10.1107/S2052520624009454/cam5004sup1.zip

Tables S1-S8, Fig. S1. DOI: 10.1107/S2052520624009454/cam5004sup2.pdf

## Figures and Tables

**Table 1:** Decomposition of the magnetic representation of the gray group *P*2_1_/*n*1′ (14.76) for ***k*** = 0 in *A*_2_*BB*′O_6_, and positions occupied by magnetic atoms in the chosen parent setting

Wyckoff position	Decomposition into irreps
2*c* (0, ½, 0)	3 mGM1+(1) ⊕ 3 mGM2+(1)
2*d* (½, 0, 0)	3 mGM1+(1) ⊕ 3 mGM2+(1)
4*e* (*x*, *y*, *z*)	3 mGM1+(1) ⊕ 3 mGM1−(1) ⊕ 3 mGM2+(1) ⊕ 3 mGM2−(1)

**Table 2:** Magnetic structure of Ho_2_MnCoO_6_ at 10 K (**FM1**) Atomic parameters and symmetry operations are given in a non-standard setting of the MSG 14.79. The transformation to a standard setting is specified.

Compound	Ho_2_MnCoO_6_	
Parent space group	*P*2_1_/*n* (No. 14) (**a**, **b**, −**a** + **c**; 0, 0, 0)	
Propagation vector(s)	(0, 0, 0)	
Transformation from parent basis to the one used	(**a**, **b**, **c**; 0, 0, 0)	
MSG symbol	*P*2_1_′/*n*′	
MSG number	14.79	
Transformation from basis used to standard setting of MSG	(**a**, **b**, −**a**+**c**; 0, 0, 0)	
Magnetic point group	2′/*m*′ (5.5.16)	
Unit-cell parameters (Å, °)	*a* = 5.21923, *b* = 5.56359, *c* = 7.45218, β = 89.77	
MSG symmetry operations	*x*, *y*, *z*, +1	{1 | 0, 0, 0}
	−*x*, −*y*, −*z*, +1	{−1 | 0, 0, 0}
	−*x* + ½, *y* + ½, −*z* + ½, −1	{2′_010_ | ½, ½, ½}
	*x* + ½, −*y* + ½, *z* + ½, −1	{m′_010_ | ½, ½, ½}
Positions of magnetic atoms	Co1 Co 0.50000 0.00000 0.00000	
	Mn1 Mn 0.00000 0.50000 0.00000	
Positions of nonmagnetic atoms	Ho1 Ho 0.0194 0.0724 0.2509	
	O1 O 0.2991 0.3157 0.0518	
	O2 O 0.3198 0.2923 0.4451	
	O3 O 0.6047 0.9667 0.2550	
Magnetic atoms, magnetic moments components of magnetic atoms, their symmetry constraints and moment magnitudes (μ_B_)	Co1 0.8 (1) 0.0 −2.80 (4) (m_*x*_, m_*y*_, m_*z*_) 2.92 (4)†	
	Mn1 0.8 (1) 0.0 −2.80 (4) (m_*x*_, m_*y*_, m_*z*_) 2.92 (4)	

† Co^2+^ and Mn^4+^ moments are constrained to have the same value.

**Table 3:** Relationship between the *P*2_1_′/*n*′ (No. 14.79) magnetic structures of *A*_2_Mn*B*′O_6_ (*A*_2_*B*′ = La_2_Co, LaTbCo, Tb_2_Co, Tb_2_Ni, Y_2_Co, Ho_2_Co, Er_2_Co, Tm_2_Co) samples and their parent paramagnetic phase As an example, the values of the amplitudes of the magnetic modes are given only for Er_2_MnCoO_6_ at 2 K (see also its magnetic structure in Table S5). For each magnetic mode, the base vector and observed amplitudes C_*n*_ (in μ_B_, *n* = 1, 2, 3, …) in the ground state of Er_2_MnCoO_6_ are indicated.

	*A*_2_MnCoO_6_ (amplitudes correspond to *A* = Er)	
Primary irrep label with dimension	mGM2+(3)	
Description of the primary irrep	mGM2+:	
	{1 | 0, 0, 0}: 1	
	{−1 | 0, 0, 0}: 1	
	{2′_010_ | ½, ½, ½}: −1	
	{m′_010_ | ½, ½, ½}: −1	
		
Description of primary mode(s) and amplitude(s) Definition of the mode(s) *P*2_1_/*n*[0,0,0]mGM2+(*a*)	mGM2+, mode 1: [*B*]Ag_1(*a*) or [*A*]*A*_1(*a*)	
	Mn (−1, 0, −1)	C1 = 1.94
	*B*′ (−1, 0, −1)	C1 = 1.94
	*A* (−1, 0, −1)	C1′ = 2.30

	mGM2+, mode 2: [*B*]Ag_2(*a*) or [*A*]*A*_2(*a*)	
	Mn (−1, 0, 0.49)	C2 = 2.34
	*B*′ (−1, 0, 0.49)	C2 = 2.34
	*A* (−1, 0, 0.49)	C2′ = 3.45

	mGM2+, mode 3: [*B*]Ag_3(*a*) or [*A*]*A*_3(*a*)	
	Mn (0, 1, 0)	C3 = 0.0
	*B*′ (0, 1, 0)	C3 = 0.0
	*A* (0, 1, 0)	C3′ = 0.0
Secondary irrep(s) label(s)	Not allowed	

C3 = 0 (not detected) for all samples except Y_2_CoMnO_6_. C*n*′ (*n* = 1,2,3) only active for magnetic *A* atoms. C3′ = 0 (not detected) for Er_2_MnCoO_6_ but active for the **FM3** phase in Tb_2_MnNiO_6_, LaTbMnCoO_6_ or Tm_2_MnCoO_6_.

**Table 4:** Magnetic structure of Lu_2_MnCoO_6_ at 2 K (E type) Atomic parameters and symmetry operations are given in a non-standard setting of the MSG No. 4.10. The transformation to a standard setting is specified.

Compound	Lu_2_MnCoO_6_	
Parent space group	*P*2_1_/*n* (No. 14) (**a**, **b**, −**a** + **c**; 0, 0, 0)	
Propagation vector(s)	(0, 0, ½)	
Transformation from parent basis to the one used	(**a**, **b**, 2**c**; 0, 0, 0)	
MSG symbol	*P*_*a*_2_1_	
MSG number	4.10	
Transformation to standard setting of MSG	(2**a** − **c**, **b**, **a**; −¼, 0, 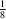 ) or (−**c**, **b**, **a**; ¼, 0, 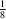 )	
Magnetic point group	21′ (No. 3.2.7)	
Unit-cell parameters (Å, °)	*a* = 5.1624, *b* = 5.5475, *c* = 14.8148, β = 89.59	
MSG symmetry operations	*x*, *y*, *z*, +1	{1|0,0,0}
	½ − *x*, ½ + *y*, ¼ − *z*, +1	{2_010_ | ½, ½, ¼}
MSG symmetry centering operations	*x*, *y*, *z* + ½, −1	{1′ | 0, 0, ½}
	½ − *x*, ½ + *y*, 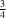 − *z*, −1	{2′_010_ | ½, ½, 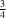 }
Positions of magnetic atoms	Co1 Co 0.75000 0.00000 0.62500	
	Mn1 Mn 0.75000 0.00000 0.37500	
Positions of nonmagnetic atoms	Lu_1 Lu 0.2294 0.0744 0.7502	
	Lu_2 Lu 0.2706 0.9256 0.4998	
	O1_1 O 0.9494 0.3203 0.6519	
	O1_2 O 0.5506 0.6797 0.5981	
	O2_1 O 0.9244 0.2964 0.8450	
	O2_2 O 0.5756 0.7036 0.4050	
	O3_1 O 0.3670 0.4559 0.74555	
	O3_2 O 0.1330 0.5441 0.50445	
Magnetic atoms, magnetic moments components of magnetic atoms, their symmetry constraints and moment magnitudes (μ_B_)	Co1 0.47 (3) 0.0 1.28 (2) (m_*x*_, m_*y*_, m_*z*_) 1.36 (2)†	
	Mn1 0.47 (3) 0.0 1.28 (2) (m_*x*_, m_*y*_, m_*z*_) 1.36 (2)	

† Co^2+^ and Mn^4+^ moments are constrained to have the same value.

**Table 5:** Relationship between the E-type magnetic structure of *A*_2_MnCoO_6_ (*A* = Yb or Lu) compounds and their parent paramagnetic phase For each magnetic mode, the base vector and observed amplitudes C*n* (in μ_B_, *n* = 1, 2, 3, …) for each site are indicated. mA1 has dimension 3 for *B* sites but 6 for *A* sites. The nuclear *A* site is split into two orbits, *A*1 and *A*2, in the magnetic cell.

Compound	*A*_2_MnCoO_6_	
Primary irrep label with dimension	mA1(3)	
Description of the primary irrep	mA1:	
	{1 | 0, 0, 0}: (1, 0; 0, 1)	
	{2_010_ | ½, ½, ¼}: (0, −1; −1, 0)	
	{1′ | 0, 0, ½}: (1, 0; 0, 1)	
	{2′_010_ | ½, ½, ¾}: (0, −1; −1, 0)	

Description of primary mode(s) and amplitude(s) Definition of the mode(s) *P*2_1_/*n*[−1, 0, ½]mA1(*a*,−*a*)	mA1, mode 1: [*B*]Ag_1(*a*) or [*A*]*A*_1(*a*)	
	Mn (−1, 0,− ½)	C1 = 0.88 [Yb], 0.78 [Lu]
	Co (1, 0, ½)	C1 = 0.88 [Yb], 0.78 [Lu]
	*A*1 (−1, 0, −½)	C1′ = 0.27 [Yb]
	*A*2 (1, 0, ½)	C1′ = 0.27 [Yb]

	mA1, mode 2: [*B*]Ag_2(a) or [*A*]*A*_2(a)	
	Mn (−1, 0, 0.24)	C2 = 1.42 [Yb], 1.12 [Lu]
	Co (1, 0, −0.24)	C2 = 1.42 [Yb], 1.12 [Lu]
	*A*1 (−1, 0, 0.24)	C2′ = 0.19 [Yb]
	*A*2 (1, 0, −0.24)	C2′ = 0.19 [Yb]

	mA1, mode 3: [*B*]Ag_3(*a*) or [*A*]*A*_3(*a*)	
	Mn (0, 1, 0)	C3 = 0.0 [Yb], 0.0 [Lu]
	Co (0, −1, 0)	C3 = 0.0 [Yb], 0.0 [Lu]
	*A*2 (0, −1, 0)	C3′ = 0.0 [Yb]
	*A*1 (0, 1, 0)	C3′ = 0.0 [Yb]

	mA1, mode 4: [*A*]*A*_4(*a*)	
	*A*1 (1, 0, ½)	C4′ = 0.27 [Yb]
	*A*2 (−1, 0, −½)	C4′ = 0.27 [Yb]

	mA1, mode 5: [*A*]*A*_5(*a*)	
	*A*1 (1, 0, −0.24)	C5′ = 0.19 [Yb]
	*A*2 (1, 0, −0.24)	C5′ = 0.19 [Yb]

	mA1, mode 6: [*A*]*A*_6(*a*)	
	*A*1 (0, 1, 0)	C6′ = 0.0 [Yb]
	*A*2 (0, 1, 0)	C6′ = 0.0 [Yb]
Secondary irrep(s) label(s)	Not allowed	

**Table 6:** Magnetic structure of Tb_2_MnNiO_6_ at 15 K (**FM2**) Atomic parameters and symmetry operations are given in a nonstandard setting of the MSG No. 14.79. The transformation to a standard setting is specified.

Compound	Tb_2_MnNiO_6_	
Parent space group	*P*2_1_/*n* (No. 14) (**a**, **b**, −**a**+**c**; 0, 0, 0)	
Propagation vector(s)	(0, 0, 0)	
Transformation from parent basis to the one used	(**a**, **b**, **c**; 0, 0, 0)	
MSG symbol	*P*2_1_′/*n*′	
MSG number	14.79	
Transformation from basis used to standard setting of MSG	(**a**, **b**, −**a**+**c**; 0, 0, 0)	
Magnetic point group	2′/*m*′ (5.5.16)	
Unit-cell parameters (Å, °)	*a* = 5.2750, *b* = 5.5167, *c* = 7.5110, β = 89.84	
MSG symmetry operations	*x*, *y*, *z*, +1	{1 | 0, 0, 0}
	−*x*, −*y*, −*z*, +1	{−1 | 0, 0, 0}
	−*x* + ½, *y* + ½, −*z* + ½, −1	{2′_010_ | ½, ½, ½}
	*x* + ½, −*y* + ½, *z* + ½, −1	{m′_010_ | ½, ½, ½}
Positions of magnetic atoms	Ni1 Ni 0.50000 0.00000 0.00000	
	Mn1 Mn 0.50000 0.00000 0.50000	
Positions of nonmagnetic atoms	Tb1 Tb 0.0177 0.0654 0.2494	
	O1 O 0.2974 0.3110 0.0509	
	O2 O 0. 3184 0.2922 0.4500	
	O3 O 0.5978 0.9678 0.2597	
Magnetic atoms, magnetic moments components of magnetic atoms, their symmetry constraints and moment magnitudes (μ_B_)	Ni1 1.25 (4) 0.0 1.22 (4) (m_*x*_, m_*y*_, m_*z*_) 1.74 (2)†	
	Mn1 1.87 (5) 0.0 1.83 (5) (m_*x*_, m_*y*_, m_*z*_) 2.61 (2)	

† 3*d* ordered moments are constrained by m[Ni^2+^] = 2/3·m[Mn^4+^].

**Table 7:** Magnetic structure of Tb_2_MnNiO_6_ at 6 K (**FM3**) Atomic parameters and symmetry operations are given in a non-standard setting of the MSG No. 14.79. The transformation to a standard setting is specified.

Compound	Tb_2_MnNiO_6_	
Parent space group	*P*2_1_/*n* (No. 14) (**a**, **b**, −**a** + **c**; 0, 0, 0)	
Propagation vector(s)	(0, 0, 0)	
Transformation from parent basis to the one used	(**a**, **b**, **c**; 0, 0, 0)	
MSG symbol	*P*2_1_′/*n*′	
MSG number	14.79	
Transformation from basis used to standard setting of MSG	(**a**, **b**, −**a** + **c**; 0, 0, 0)	
Magnetic point group	2′/*m*′ (No. 5.5.16)	
Unit-cell parameters (Å, °)	*a* = 5.2750, *b* = 5.5165, *c* = 7.5113, β = 89.84	
MSG symmetry operations	*x*, *y*, *z*, +1	{1 | 0, 0, 0}
	−*x*, −*y*, −*z*, +1	{−1 | 0, 0, 0}
	−*x* + ½, *y* + ½, −*z* + ½, −1	{2′_010_ | ½, ½, ½}
	*x* + ½,−*y* + ½, *z* + ½, −1	{m′_010_ | ½, ½, ½}
Positions of magnetic atoms	Ni1 Ni 0.50000 0.00000 0.00000	
	Mn1 Mn 0.50000 0.00000 0.50000	
	Tb1 Tb 0.0177 0.0654 0.2494	
Positions of nonmagnetic atoms	O1 O 0.2974 0.3110 0.0509 0.9678 0.2597	
	O2 O 0. 3184 0.2922 0.4500 O3 O 0.5978	
Magnetic atoms, magnetic moments components of magnetic atoms, their symmetry constraints and moment magnitudes (μ_B_)	Ni1 1.64 (5) 0.0 0.76 (5) (m_*x*_, m_*y*_, m_*z*_) 1.81 (3)†	
	Mn1 2.46 (6) 0.0 1.15 (6) (m_*x*_, m_*y*_, m_*z*_) 2.72 (4)	
	Tb1 0.76 (4) 0.90 (4) 0.0 (m_*x*_, m_*y*_, m_*z*_) 1.18 (4)	

† 3*d* ordered moments are constrained by m[Ni^2+^] = 2/3·m[Mn^4+^].

**Table 8:** Magnetic structure of Tb_2_MnNiO_6_ at 2 K (**FM4**) Atomic parameters and symmetry operations are given in a non-standard setting of the MSG 4.9. The transformation to a standard setting is specified.

Compound	Tb_2_MnNiO_6_	
Parent space group	*P*2_1_/*n* (No. 14) (**a**, **b**, −**a**+**c**; 0, 0, 0)	
Propagation vector(s)	(0, 0, 0)	
Transformation from parent basis to the one used	(**a**, **b**, **c**; 0, 0, 0)	
MSG symbol	*P*2_1_′	
MSG number	4.9	
Transformation from basis used to standard setting of MSG	(**a**, **b**, **c**; ¼, 0, ¼)	
Magnetic point group	2′ (No. 3.3.8)	
Unit-cell parameters (Å, °)	*a* = 5.2745, *b* = 5.5164, *c* = 7.5102, β = 89.839	
MSG symmetry operations	*x*, *y*, *z*, +1	{1 | 0, 0, 0}
	−*x* + ½, *y* + ½, −*z* + ½, −1	{2′_010_ | ½, ½, ½}
Positions of magnetic atoms	Ni1 Ni 0.50000 0.00000 0.00000	
	Mn1 Mn 0.50000 0.00000 0.50000	
	Tb1 Tb 0.0177 0.0654 0.2494	
	Tb2 Tb 0.9823 0.9346 0.7506	
Positions of nonmagnetic atoms	O1_1 O 0.2974 0.3110 0.0509	
	O1_2 O 0.7026 0.6890 0.9491	
	O2_1 O 0.3184 0.2922 0.4500	
	O2_2 O 0.6816 0.7078 0.5500	
	O3_1 O 0.5978 0.9678 0.2597	
	O3_2 O 0.4022 0.0322 0.7403	
Magnetic atoms, magnetic moments components of magnetic atoms, their symmetry constraints and moment magnitudes (μ_B_)	Ni1 0.8 (2) 0.0 1.6 (1) (m_*x*_, m_*y*_, m_*z*_) 1.9 (2)†	
	Mn1 1.3 (3) 0.0 2.4 (1) (m_*x*_, m_*y*_, m_*z*_) 2.7 (2)	
	Tb1 5.84 (6) 4.7 (2) 0.0 (m_*x*_, m_*y*_, m_*z*_) 7.5 (1)‡	
	Tb2 −5.84 (6) −4.7 (2) 0.0 (m_*x*_, m_*y*_, m_*z*_) −7.5 (1)	

† 3*d* ordered moments are constrained by m[Ni^2+^] = ⅔m[Mn^4+^].

‡ 4*f* ordered moments are constrained by m[Tb1^3+^] = −m[Tb2^3+^].

**Table 9:** Relationship between the magnetic structure of the **FM4** phase of Tb_2_MnNiO_6_ (*P*2_1_′ (**a**, **b**, **c**; ¼, 0, ¼) and its parent paramagnetic phase For each magnetic mode, the base vector and observed amplitudes C*n* (in μ_B_, *n* = 1, 2, 3, …) for each site are indicated.

Compound	Tb_2_MnNiO_6_	
Parent space group	*P*2_1_/*n* (No. 14) (**a**, **b**, −**a** + **c** ; 0, 0, 0)	
Transformation from parent basis to the one used for the magnetic structure	(**a**, **b**, **c**; 0, 0, 0)	
Propagation vector	(0, 0, 0)	
Primary irrep(s) label(s) with dimension	mGM2+ (3)	
	mGM2− (3)	
Description of the primary irrep(s)	mGM2+:	
	{1 | 0, 0, 0}: 1	
	{−1 | 0, 0, 0}: 1	
	{2′_010_ | ½, ½, ½}: −1	
	{m′_010_ | ½, ½, ½}: −1	

	mGM2−:	
	{1 | 0, 0, 0}: 1	
	{−1′ | 0, 0, 0}: −1	
	{2′_010_ | 0, ½, 0}: −1	
	{m_010_ | ½, ½, ½}: 1	

Description of primary mode(s) and amplitude(s) Definition of the mode(s) *P*2_1_/*n*[0,0,0]mGM2+(*a*) *P*2_1_/*n*[0,0,0]mGM2−(*a*)	mGM2+, mode 1: [*B*]Ag_1(*a*)	
	Mn1 (−1, 0, −1)	C_1_ = 2.71
	Ni1 (−1, 0, −1)	C_1_ = 1.77

	mGM2+, mode 2: [*B*]Ag_2(*a*)	
	Mn1 (−1, 0, 0.491)	C_2_ = 0.31
	Ni1 (−1, 0, 0.491)	C_2_ = 0.26

	mGM2+, mode 3: [*B*]Ag_3(*a*)	
	Mn1 (0, 1, 0)	C_3_ = 0.0
	Ni1 (0, 1, 0)	C_3_ = 0.0

	mGM2−, mode 1: [A]A_1(*a*)	
	Tb1 (−1, 0, −1)	C_1_ = 3.35
	Tb2 (1, 0, 1)	C_1_ = 3.35

	mGM2−, mode 2: [*A*]*A*_2(*a*)	
	Tb1 (−1, 0, 0.491)	C_2_ = 4.77
	Tb2 (1, 0, −0.491)	C_2_ = 4.77

	mGM2−, mode 3: [*A*]*A*_3(*a*)	
	Tb1 (0, 1, 0)	C_3_ = 4.72
	Tb2 (0, −1, 0)	C_3_ = 4.72
Secondary irrep(s) label(s)	Not allowed	
