# Supplementary material for: Symmetry, magnetic transitions and multiferroic properties of B-site-ordered A2MnB′O6 perovskites (B′ = [Co, Ni])
Source: Acta Crystallogr B Struct Sci Cryst Eng Mater. 2024 Nov 8;80(Pt 6):665–75. doi: 10.1107/S2052520624009454 (PMC11789168; doi:10.1107/S2052520624009454)
Supplement: Supplementary file 2 [file b-80-00665-sup2.pdf]

STRUCTURAL SCIENCE
CRYSTAL ENGINEERING
MATERIALS

Volume 80 (2024)

Supporting information for article:

Symmetry, magnetic transitions and multiferroic properties of *B*-site-ordered *A*2Mn*B′*O6 perovskites (*B′* = [Co, Ni])

Jose Luis Garcia-Muñoz, Xiaodong Zhang, Gloria Subías and Javier Blasco

Supplementary Material

Supplementary Material (SM) for

Symmetry, magnetic transitions and multiferroic properties of B site ordered $A_2MnB'O_6$ perovskites (B'=[Co, Ni])

Jose Luis Garcia-Muñoz[a]*, Xiaodong Zhang[a], Gloria Subías[b,c] and Javier Blasco[b,c]

[a]Instituto de Ciencia de Materiales de Barcelona, ICMAB-CSIC, Carrer dels Til·lers s/n. Campus de la UAB, Bellaterra, Catalunya, 08193, Spain

[b]Instituto de Nanociencia y Materiales de Aragón (INMA), CSIC-Universidad de Zaragoza, 50009 Zaragoza 50009, Spain

[c]Departamento de Física de la Materia Condensada, Universidad de Zaragoza, C/ Pedro Cerbuna 12, 50009 Zaragoza, Spain

*Corresponding author. Email: garcia.munoz@icmab.es

This PDF file includes:

- Tables S1 to S8

- Figure S1

Table S1 Refined structural parameters (lattice, fractional coordinates and temperature factors) and reliability factors from neutron patterns collected at room temperature for $A_2MnB'O_6$. (A=La, LaTb, Tb and Y and B'=Co or Ni). The space group is *P2₁/n* and the Wyckoff positions are indicated for each atom (coordinates are given in the non-standard setting *P2₁/n* (**a**,**b**,**-a+c**;0,0,0)). Patterns were collected at D1B for A=La, Tb, and at D2B for A=Tb and Y.

Compound	La_2MnCoO_6	$LaTbMnCoO_6$	Tb_2MnCoO_6	Tb_2MnNiO_6	Y_2MnCoO_6
a(Å)	5.5617(6)	5.4356(4)	5.28149(7)	5.2719(2)	5.22341(5)
b (Å)	5.5227(4)	5.5602(4)	5.58735(7)	5.5377(2)	5.57083(5)
c (Å)	7.8186(9)	7.7098(6)	7.51978(9)	7.5278(2)	7.46105(8)
β (º)	89.90(1)	89.87(1)	89.878(1)	89.839(3)	89.761(1)
Volume (Å^3)	240.16(5)	233.01(3)	221.905(5)	219.77(1)	217.105(4)
A(4e): x	0.0010(14)	0.0089(12)	0.0162(1)	0.0177(3)	0.0182(2)
y	0.0230(8)	0.0482(8)	0.0676(1)	0.0654(3)	0.0722(2)
z	0.2442(17)	0.2506(32)	0. 2503 (2)	0.2494(3)	0.2508(2)
B(Å^2)	0.98(17)	0.51(21)	1.36(1)	0.68(4)	0.63(2)
Mn(2c): B(Å^2)	0.98(17)	0.51(21)	2.07(7)	1.37(5)	0.94(9)
B'(2d): B(Å^2)	0.98(17)	0.51(21)	2.07(7)	1.37(5)	0.75(9)
ASD(%)	18(2)	42(1)	6.4(8)	1.0(8)	8.1(3)
O1(4e): x	0.2553(21)	0.2859(32)	0.2958(3)	0.2974(4)	0.2986(3)
y	0.2825(24)	0.2963(31)	0.3116(3)	0.3109(5)	0.3154(4)
z	0.0255(13)	0.0443(27)	0.0498(2)	0.0509(4)	0.0512(2)
B(Å^2)	0.98(17)	0.51(21)	1.54(1)	1.46(6)	0.72(3)
O2(4e): x	0.2930(26)	0.2991(29)	0.3146(2)	0.3184(5)	0.3185(3)
y	0.2699(24)	0.2903(32)	0.2927(2)	0.2922(5)	0.2933(3)
z	0.4580(10)	0.4581(28)	0.4484(2)	0.4500(4)	0.4442(3)
B(Å^2)	0.98(17)	0.51(21)	1.54(1)	1.25(6)	0.74(4)
O3(4e): x	0.5719(14)	0.5859(12)	0.5999(2)	0.5978(4)	0.6060(2)
y	0.9951(14)	0.9787(10)	0.9679(2)	0.9678(4)	0.9653(3)
z	0.2579(33)	0.2505(25)	0.2565(2)	0.2597(3)	0.2564(3)
B(Å^2)	0.98(17)	0.51(21)	1.54(1)	1.55(5)	0.73(3)
R_B	2.4	2.6	1.1	4.4	3.3
R_{wp}	3.3	2.3	2.0	4.0	3.9
χ^2	1.6	1.7	1.2	1.5	3.0

Table S2 Refined structural parameters (lattice, fractional coordinates, temperature factors, bond lengths and bond angles) and reliability factors from neutron patterns collected at D2B and at 295 K for A_2MnCoO_6. (A=Ho, Er, Tm, Yb, and Lu). The space group is *P2₁/n* and the Wyckoff positions are indicated for each atom (coordinates are given in the non-standard setting *P2₁/n* (**a**,**b**,**-a+c**;0,0,0)).

Compound	Ho_2MnCoO_6	Er_2MnCoO_6	Tm_2MnCoO_6	Yb_2MnCoO_6	Lu_2MnCoO_6
a (Å)	5.22487(6)	5.20577(7)	5.18998(6)	5.17698(4)	5.1603(1)
b (Å)	5.56795(6)	5.56242(7)	5.55221(6)	5.54841(4)	5.5421(1)
c (Å)	7.46649(8)	7.44804(9)	7.43221(8)	7.41821 (6)	7.4127(1)
β (º)	89.749(1)	89.713(1)	89.713(1)	89.648(1)	89.622(1)
Volume (Å^3)	217.212(4)	215.668(5)	214.162(4)	213.076(3)	211.992(4)
A(4e): x	0.0180(2)	0.0198(3)	0.0204(3)	0.0215(1)	0.0207(3)
y	0.0713(2)	0.0728(2)	0.0740(2)	0.0750(1)	0.0749(3)
z	0.2509(3)	0.2513(3)	0.2515(3)	0.2513(1)	0.2513(2)
B(Å^2)	0.37(2)	0.63(2)	0.35(2)	0.38(1)	0.39(2)
Mn(2c): B(Å^2)	0.75(12)	0.84(12)	0.58(9)	0.42(8)	0.26(9)
Co(2d): B(Å^2)	0.45(20)	0.93(12)	1.24(16)	0.70(15)	0.99(16)
ASD(%)	6.3(5)	5.4 (5)	6.5 (8)	5.8(8)	4.4(8)
O1(4e): x	0.2989(4)	0.2984(5)	0.2995(4)	0.2998(3)	0.3009(4)
y	0.3164(5)	0.3169(5)	0.3169(4)	0.3190(4)	0.3189(4)
z	0.0510(3)	0.0526(3)	0.0532(2)	0.0547(2)	0.0554(3)
B(Å^2)	0.75(4)	0.80(4)	0.48(3)	0.54(3)	0.46(3)
O2(4e): x	0.3178(4)	0.3206(4)	0.3219(3)	0.3248(4)	0.3246(4)
y	0.2938(4)	0.2931(5)	0.2937(4)	0.2945(4)	0.2963(4)
z	0.4441(3)	0.4434(3)	0.4412(3)	0.4402(3)	0.4391(3)
B(Å^2)	0.62(4)	0.90(5)	0.54(4)	0.53(3)	0.58(4)
O3(4e): x	0.6053(3)	0.6081(3)	0.6116(3)	0.6138(3)	0.6164(3)
y	0.9655(3)	0.9645(3)	0.9617(3)	0.9582(3)	0.9570(3)
z	0.2563(3)	0.2563(3)	0.2570(3)	0.2573(3)	0.2571(3)
B(Å^2)	0.70(3)	0.82(3)	0.55(3)	0.54(3)	0.46(3)
R_B	3.18	3.01	3.09	3.33	3.82
R_{wp}	2.88	2.99	2.76	3.29	4.11
χ^2	1.93	1.75	1.81	2.64	1.55

Table S3 Magnetic structure of Tb_2MnCoO_6 at 15 K. Atomic parameterss and symmetry operations are given in a non-standard setting of the MSG 14.79. The transformation to a standard setting is specified.

**Compound**	**Tb_2MnCoO_6** 15 K	
Parent space group	*$P2_1/n$* (N. 14)	
Propagation vector(s)	(0, 0, 0)	
Transformation from parent basis to the one used	(**a**,**b**,**c**;0,0,0)	
MSG symbol	*$P2_1'/n'$*	
MSG number	14.79	
Transformation from basis used to standard setting of MSG	(**a**,**b**,**-a+c**;0,0,0)	
Magnetic point group	*2'/m'* (5.5.16)	
Unit cell parameters (Å)	a=5.28242 α=90º<br>b=5.56091 β =89.873º<br>c=7.49263 γ =90º	
MSG symmetry operations	x,y,z,+1<br>-x,-y,-z,+1<br>-x+½,y+½,-z+½,-1<br>x+½,-y+½,z+½,-1	{1|0,0,0}<br>{-1|0,0,0}<br>{$2'_{010}$|½,½,½}<br>{m'_{010}|½,½,½}
Positions of magnetic atoms	Co1 Co 0.50000 0.00000 0.00000<br>Mn1 Mn 0.50000 0.00000 0.50000	
Positions of non-magnetic atoms	Tb1 Tb 0.0162 0.0676 0.2503<br>O1 O 0.2958 0.3116 0.0498<br>O2 O 0.3146 0.2927 0.4484<br>O3 O 0. 5999 0.9679 0.2565	
Magnetic moments components (μB) of magnetic atoms, their symmetry constraints and moment magnitudes.	Co1 -0.56(8) 0.0 2.73(2) (mx,my,mz) 2.79(2) *<br>Mn1 -0.56(8) 0.0 2.73(2) (mx,my,mz) 2.79(2)	

*Co^{2+} and Mn^{4+} moments are constrained to have the same value.

Table S4 Magnetic structure of Y_2MnCoO_6 at 2 K. Atomic parameterss and symmetry operations are given in a non-standard setting of the MSG 14.79. The transformation to a standard setting is specified.

**Compound**	**Y_2MnCoO_6** 2 K	
Parent space group	$P2_1/n$ (N. 14)	
Propagation vector(s)	(0, 0, 0)	
Transformation from parent basis to the one used	(**a**,**b**,**c**;0,0,0)	
MSG symbol	*P2₁'/n'*	
MSG number	14.79	
Transformation to standard setting of MSG	(**a**,**b**,**-a+c**;0,0,0)	
Magnetic point group	*2'/m'* (5.5.16)	
Unit cell parameters (Å)	a=5.21683 α=90º<br>b=5.56803 β =89.76º<br>c=7.44896 γ =90º	
MSG symmetry operations	x,y,z,+1<br>-x,-y,-z,+1<br>-x+½,y+½,-z+½,-1<br>x+½,-y+½,z+½,-1	{1|0,0,0}<br>{-1|0,0,0}<br>{2'$_{010}$|½,½,½}<br>{m'$_{010}$|½,½,½}
Positions of magnetic atoms	Co1 Co 0.50000 0.00000 0.00000<br>Mn1 Mn 0.00000 0.50000 0.00000	
Positions of non-magnetic atoms	Y1 Y 0.0193 0.0729 0.2512<br>O1 O 0.2986 0.3154 0.0515<br>O2 O 0.3197 0.2924 0.4443<br>O3 O 0.6059 0.9661 0.2557	
Magnetic moments components (μB) of magnetic atoms, their symmetry constraints and moment magnitudes.	Co1 0.85(6) 0.33(3) 2.78(2) (mx,my,mz) 2.92(2)<br>Mn1 0.85(6) 0.33(3) 2.78(2) (mx,my,mz) 2.92(2)	

*Co^{2+} and Mn^{4+} moments are constrained to have the same value.

Table S5 Magnetic structure of Er_2MnCoO_6 at 2 K. Atomic parameterss and symmetry operations are given in a non-standard setting of the MSG 14.79. The transformation to a standard setting is specified.

Compound	**Er_2MnCoO_6** 2 K	
Parent space group	*P2₁/n* (N. 14)	
Propagation vector(s)	(0, 0, 0)	
Transformation from parent basis to the one used	(**a**,**b**,**c**;0,0,0)	
MSG symbol	*P2₁'/n'*	
MSG number	14.79	
Transformation from basis used to standard setting of MSG	(**a**,**b**,**-a+c**;0,0,0)	
Magnetic point group	*2'/m'* (5.5.16)	
Unit cell parameters (Å)	a=5.19974 α=90º<br>b=5.55720 β =89.71º<br>c=7.43423 γ =90º	
MSG symmetry operations	x,y,z,+1<br>-x,-y,-z,+1<br>-x+½,y+½,-z+½,-1<br>x+½,-y+½,z+½,-1	{1|0,0,0}<br>{-1|0,0,0}<br>{2'₀₁₀|½,½,½}<br>{m'₀₁₀|½,½,½}
Positions of magnetic atoms	Co1 Co 0.50000 0.00000 0.00000<br>Mn1 Mn 0.00000 0.50000 0.00000<br>Er1 Er 0.0213 0.0746 0.2501	
Positions of non-magnetic atoms	O1 O 0.2999 0.3171 0.0533<br>O2 O 0.3221 0.2909 0.4436<br>O3 O 0.6092 0.9645 0.2547	
Magnetic moments components (μB) of magnetic atoms, their symmetry constraints and moment magnitudes.	Co1 0.8(1) 0.0 -2.92(6) (mx,my,mz) 3.02(6)<br>Mn1 0.8(1) 0.0 -2.92(6) (mx,my,mz) 3.02(6)<br>Er1 -1.5(1) 0.0 3.85(6) (mx,my,mz) 4.12(6)	

*Co^{2+} and Mn^{4+} moments are constrained to have the same value.

Table S6 Magnetic structure of LaTbMnCoO6 at 2 K. Atomic parameterss and symmetry operations are given in a non-standard setting of the MSG 14.79. The transformation to a standard setting is specified.

<table>
<tr><td>Compound</td><td colspan="2">$LaTbMnCoO_6$ 2 K</td></tr>
<tr><td>Parent space group</td><td colspan="2">$P2_1/n$ (N. 14)</td></tr>
<tr><td>Propagation vector(s)</td><td colspan="2">(0, 0, 0)</td></tr>
<tr><td>Transformation from parent basis to the one used</td><td colspan="2">(a,b,c;0,0,0)</td></tr>
<tr><td>MSG symbol</td><td colspan="2">$P2_1'/n'$</td></tr>
<tr><td>MSG number</td><td colspan="2">14.79</td></tr>
<tr><td>Transformation from basis used to standard setting of MSG</td><td colspan="2">(a,b,-a+c;0,0,0)</td></tr>
<tr><td>Magnetic point group</td><td colspan="2">2'/m' (5.5.16)</td></tr>
<tr><td>Unit cell parameters (Å)</td><td colspan="2">a=5.43301 α=90º
b=5.55457 β =89.97º
c=7.69265 γ =90º</td></tr>
<tr><td>MSG symmetry operations</td><td>x,y,z,+1
-x,-y,-z,+1
-x+½,y+½,-z+½,-1
x+½,-y+½,z+½,-1</td><td>{1|0,0,0}
{-1|0,0,0}
{$2'_{010}$|½,½,½}
{m'_{010}|½,½,½}</td></tr>
<tr><td>Positions of magnetic atoms</td><td colspan="2">Co1 Co(Mn) 0.50000 0.00000 0.00000 0.6(0.4)
Mn1 Mn(Co) 0.00000 0.50000 0.00000 0.6(0.4)
Tb1 Tb 0.0120 0.0519 0.2522 0.5*</td></tr>
<tr><td>Positions of non-magnetic atoms</td><td colspan="2">La1 La 0.0120 0.0519 0.2522 0.5*
O1 O 0.2919 0.2884 0.0484 1
O2 O 0.2908 0.2938 0.4626 1
O3 O 0.5872 0.9799 0.2401 1</td></tr>
<tr><td>Magnetic moments components (μB) of magnetic atoms, their symmetry constraints and moment magnitudes.</td><td colspan="2">Co1 1.7(1) 0.0 0.6(1) (mx,my,mz) 1.83(7)#
Mn1 1.7(1) 0.0 0.6(1) (mx,my,mz) 1.83(7)
Tb1 1.5(1) 2.04(8) 0.0 (mx,my,mz) 2.53(8)</td></tr>
</table>

*Co^{2+} and Mn^{4+} moments are constrained to have the same value.

Table S7 Magnetic structure of Tm_2MnCoO_6 at 2 K. Atomic parameterss and symmetry operations are given in a non-standard setting of the MSG 14.79. The transformation to a standard setting is specified.

Compound	**Tm_2MnCoO_6** 2 K	
Parent space group	*$P2_1/n$* (N. 14)	
Propagation vector(s)	(0, 0, 0)	
Transformation from parent basis to the one used	(**a**,**b**,**c**;0,0,0)	
MSG symbol	*$P2_1'/n'$*	
MSG number	14.79	
Transformation from basis used to standard setting of MSG	(**a**,**b**,**-a+c**;0,0,0)	
Magnetic point group	*2'/m'* (5.5.16)	
Unit cell parameters (Å)	a=5.18943 α=90º<br>b=5.54304 β =89.67º<br>c=7.41935 γ =90º	
MSG symmetry operations	x,y,z,+1<br>-x,-y,-z,+1<br>-x+½,y+½,-z+½,-1<br>x+½,-y+½,z+½,-1	{1|0,0,0}<br>{-1|0,0,0}<br>{2'$_{010}$|½,½,½}<br>{m'$_{010}$|½,½,½}
Positions of magnetic atoms	Co1 Co 0.50000 0.00000 0.00000<br>Mn1 Mn 0.00000 0.50000 0.00000<br>Tm1 Tm 0.0208 0.0732 0.2520	
Positions of non-magnetic atoms	O1 O 0.3005 0.3173 0.0541<br>O2 O 0.3222 0.2934 0.4423<br>O3 O 0.6123 0.9626 0.2561	
Magnetic moments components (μB) of magnetic atoms, their symmetry constraints and moment magnitudes.	Co1 1.1(1) 0.0 -2.60(6) (mx,my,mz) 2.83(6)<br>Mn1 1.1(1) 0.0 -2.60(6) (mx,my,mz) 2.83(6)<br>Tm1 -1.38(7) 0.92(4) 1.42(5) (mx,my,mz) 2.18(7)	

*Co^{2+} and Mn^{4+} moments are constrained to have the same value.

Table S8 Magnetic structure of Yb_2MnCoO_6 at 2 K. Atomic parameterss and symmetry operations are given in a non-standard setting of the MSG 4.10. The transformation to a standard setting is specified.

**Compound**	**Yb_2MnCoO_6** 2 K	
Parent space group	*P2₁/n* (N. 14)	
Propagation vector(s)	(0, 0, ½)	
Transformation from parent basis to the one used	(**a**,**b**,2**c**;0,0,0)	
MSG symbol	P_a2_1	
MSG number	4.10	
Transformation to standard setting of MSG	(2**a**-**c**,**b**,**a**;-1/4,0,1/8) or (-**c**,**b**,**a**; 1/4,0,1/8)	
Magnetic point group	*21'* (3.2.7)	
Unit cell parameters (Å)	a=5.1777 α=90º<br>b=5.5471 β =89.65º<br>c=14.8147 γ =90º	
MSG symmetry operations	x,y,z,+1<br>½-x, ½ +y, ¼ -z, +1	{1|0,0,0}<br>{2_{010}|½, ½,¼}
MSG symmetry centering operations	x,y,z, -1<br>½ -x, ½+y, ¾-z, -1	{1'|0,0,½}<br>{$2'_{010}$|½,½,¾}
Positions of magnetic atoms	Co1 Co 0.75000 0.00000 0.62500<br>Mn1 Mn 0.75000 0.00000 0.37500<br>Yb_2 Yb 0.27100 0.92550 0.49930	
Positions of non-magnetic atoms	Yb_1 Yb 0.2290 0.0745 0.7507<br>O1_1 O 0.9512 0.3215 0.6513<br>O1_2 O 0.5488 0.6785 0.5987<br>O2_1 O 0.9232 0.2955 0.8451<br>O2_2 O 0.5768 0.7045 0.4049<br>O3_1 O 0.3642 0.4565 0.74455<br>O3_2 O 0.1358 0.5435 0.50545	
Magnetic moments components (μB) of magnetic atoms, their symmetry constraints and moment magnitudes.	Co1 0.66(5) 0.0 1.53(4) (mx,my,mz) 1.66(4)<br>Mn1 0.66(5) 0.0 1.53(4) (mx,my,mz) 1.66(4)<br>Yb_2 0.0 0.0 0.65(3) (mx,my,mz) 0.65(3)	

*Co^{2+} and Mn^{4+} moments are constrained to have the same value.

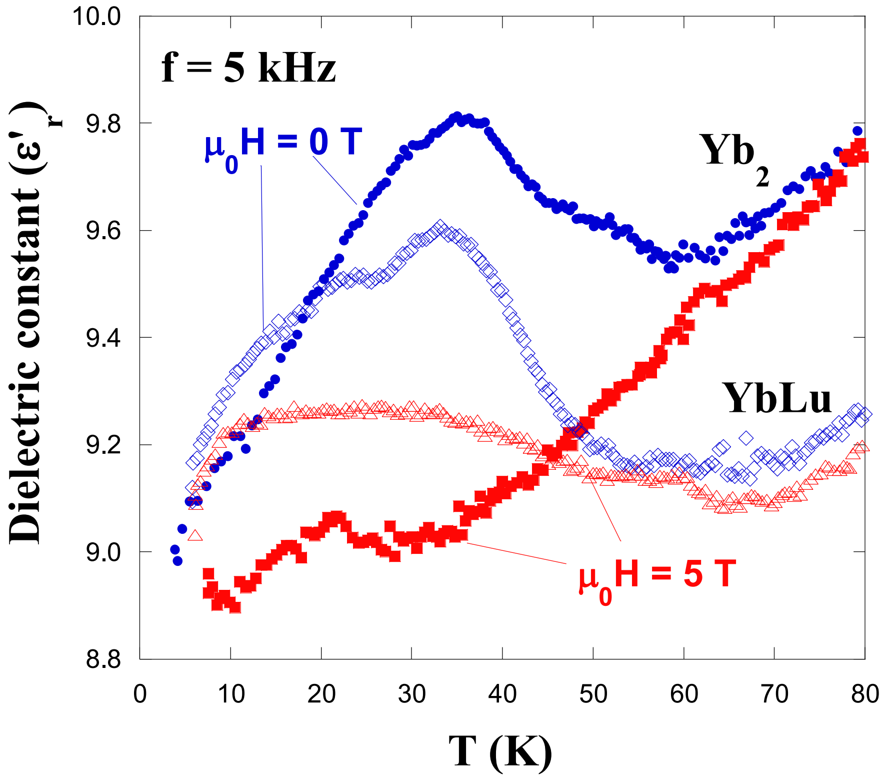

Figure S1 Temperature dependence of the real part of the relative dielectric permittivity for Yb_2MnCoO_6 and $YbLuMnCoO_6$ with and without an external field of 5 T.
